# Deleting *Mecp2* from the cerebellum rather than its neuronal subtypes causes a delay in motor learning in mice

**DOI:** 10.7554/eLife.64833

**Published:** 2021-01-26

**Authors:** Nathan P Achilly, Ling-jie He, Olivia A Kim, Shogo Ohmae, Gregory J Wojaczynski, Tao Lin, Roy V Sillitoe, Javier F Medina, Huda Y Zoghbi

**Affiliations:** 1 Jan and Dan Duncan Neurological Research Institute, Texas Children’s HospitalHoustonUnited States; 2 Program in Developmental Biology, Baylor College of MedicineHoustonUnited States; 3 Medical Scientist Training Program, Baylor College of MedicineHoustonUnited States; 4 Department of Human and Molecular Genetics, Baylor College of MedicineHoustonUnited States; 5 Howard Hughes Medical Institute, Baylor College of MedicineHoustonUnited States; 6 Department of Neuroscience, Baylor College of MedicineHoustonUnited States; 7 Department of Pathology and Immunology, Baylor College of MedicineHoustonUnited States; 8 Department of Neurology, Baylor College of MedicineHoustonUnited States; 9 Department of Pediatrics, Baylor College of MedicineHoustonUnited States; Oregon Health and Science UniversityUnited States; Oregon Health and Science UniversityUnited States

**Keywords:** Rett syndrome, cerebellum, motor learning, MeCP2, Mouse

## Abstract

Rett syndrome is a devastating childhood neurological disorder caused by mutations in *MECP2*. Of the many symptoms, motor deterioration is a significant problem for patients. In mice, deleting *Mecp2* from the cortex or basal ganglia causes motor dysfunction, hypoactivity, and tremor, which are abnormalities observed in patients. Little is known about the function of *Mecp2* in the cerebellum, a brain region critical for motor function. Here we show that deleting *Mecp2* from the cerebellum, but not from its neuronal subtypes, causes a delay in motor learning that is overcome by additional training. We observed irregular firing rates of Purkinje cells and altered heterochromatin architecture within the cerebellum of knockout mice. These findings demonstrate that the motor deficits present in Rett syndrome arise, in part, from cerebellar dysfunction. For Rett syndrome and other neurodevelopmental disorders, our results highlight the importance of understanding which brain regions contribute to disease phenotypes.

## Introduction

Loss-of-function mutations in *MECP2* (human gene) cause a severe childhood disorder called Rett syndrome (Amir et al., 1999). After a period of normal development, patients lose previously acquired milestones and develop debilitating neurological deficits (Hagberg et al., 1983; Neul et al., 2014). Of these symptoms, motor deterioration is a significant problem for patients and manifests as ataxia, apraxia, hypotonia, and spasticity (Neul et al., 2014; Sandweiss et al., 2020). In mice, the complete loss of *Mecp2* (mouse gene) causes deficits in motor coordination and motor learning, hind limb clasping, hypoactivity, and tremors that mimic those seen in patients (Chen et al., 2001; Guy et al., 2001; Pelka et al., 2006). Furthermore, mice that conditionally lack *Mecp2* in the cortex or basal ganglia partially replicate these Rett-like impairments (Chen et al., 2001; Gemelli et al., 2006; Su et al., 2015), suggesting that forebrain dysfunction contributes to the motor deficits seen in patients. However, other brain regions such as the cerebellum also control motor activity (Bostan and Strick, 2018) and may contribute to the complex, wide-ranging motor phenotypes of Rett syndrome.

The cerebellum contains approximately 75% of all neurons in the brain (Lange, 1975; Sarko et al., 2009) and integrates sensory inputs in order to fine-tune motor output (Manto et al., 2012). This function is critical for motor coordination and motor learning as impairments in the cerebellar circuitry cause ataxia, dystonia, and tremor (White et al., 2016; Bostan and Strick, 2018; Darmohray et al., 2019; Machado et al., 2020). The cerebellum also contributes to non-motor behaviors such as social interaction, reward, and memory (Wagner et al., 2017; Carta et al., 2019; McAfee et al., 2019; Kelly et al., 2020). Interestingly, some of the motor and non-motor symptoms of Rett syndrome overlap with those of conditions that perturb cerebellar function such as spinocerebellar ataxias, tumors, and strokes (Schmahmann, 2004; Sokolov, 2018; Sandweiss et al., 2020). Therefore, we hypothesized that *Mecp2* deficiency disrupts cerebellar function and leads to motor phenotypes similar to those seen in Rett syndrome. To test this hypothesis, we deleted *Mecp2* from the cerebellum and discovered that cerebellar knockout (KO) animals had deficits in motor learning that were overcome with additional training. This motor learning delay was accompanied by irregular firing patterns of Purkinje cells and a reduction in H3K9me3 levels in heterochromatic foci of granule cells, Purkinje cells, and molecular layer interneurons. These data indicate that *Mecp2* deficiency in the cerebellum is consequential and contributes to the motor dysfunction seen in Rett syndrome.

## Results

### Deleting *Mecp2* from all major neuronal subtypes in the cerebellum causes a delay in motor learning

**Figure 1.**
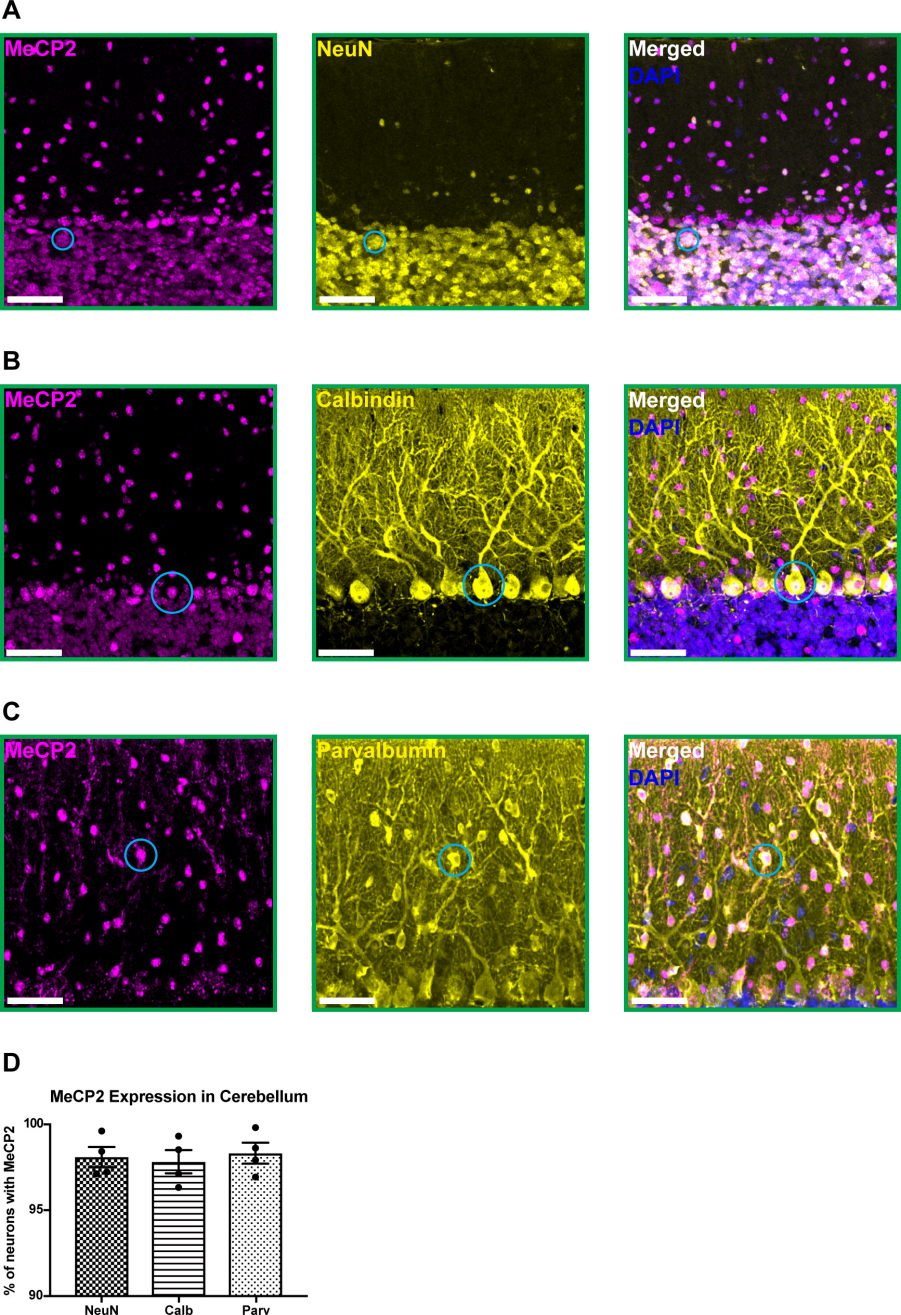
MeCP2 is expressed in cerebellar neurons of 6-month-old wild-type mice. (**A–C**) MeCP2 (magenta) staining in NeuN+ neurons (yellow) in the granular layer (**A**: solid cyan circle), Calbindin+ neurons (yellow) in the Purkinje cell layer (**B**: solid cyan circle), and Parvalbumin+ neurons (yellow) in the molecular layer (**C**: solid cyan circle). Scale bar, 25 µm. (**D**) Quantification of the percentage of NeuN+, Calbindin+, and Parvalbumin+ neurons that express MeCP2. N = 4 biologically independent mice per group. Data are presented as mean ± s.e.m. Figure 1—source data 1.Related to Figure 1.

To confirm that MeCP2 (mouse protein) is expressed in cerebellar neurons, we performed immunostaining for MeCP2 and a variety of neuron-specific makers in 6-month-old wild-type mice. MeCP2 was expressed in granule cells, Purkinje cells, and molecular layer interneurons (Figure 1A–D). This suggests that MeCP2 may contribute to the function of these neuronal populations.

To test this, we conditionally deleted *Mecp2* either from all major cell types in the cerebellum using *En1^Cre^* mice or individually from granule cells, Purkinje cells, and cerebellar inhibitory neurons (Purkinje cells and molecular layer interneurons) using *Atoh1^Cre^*, *Pcp2^Cre^*, and *Ptf1a^Cre^* mice, respectively (Hoshino et al., 2005; Joyner and Zervas, 2006; Hashimoto and Hibi, 2012; Sługocka et al., 2017). We verified the recombination efficiency using *Rosa26^lsl-tdTomato^* reporter mice and found that granule cells, Purkinje cells, and molecular layer interneurons expressed tdTomato (Figure 2—figure supplement 1A–C; Figure 2—figure supplement 2A–B,D–E,G–H). We crossed Cre-expressing and *Mecp2^flox/+^* animals to generate *Mecp2* conditional KO mice and littermate controls (WT, Flox, and Cre) and confirmed that MeCP2 was deleted from the cerebellum or from granule cells, Purkinje cells, and cerebellar inhibitory neurons (Purkinje cells and molecular layer interneurons) (Figure 2—figure supplement 1D,E; Figure 2—figure supplement 2C,F,I).

**Figure 2.**
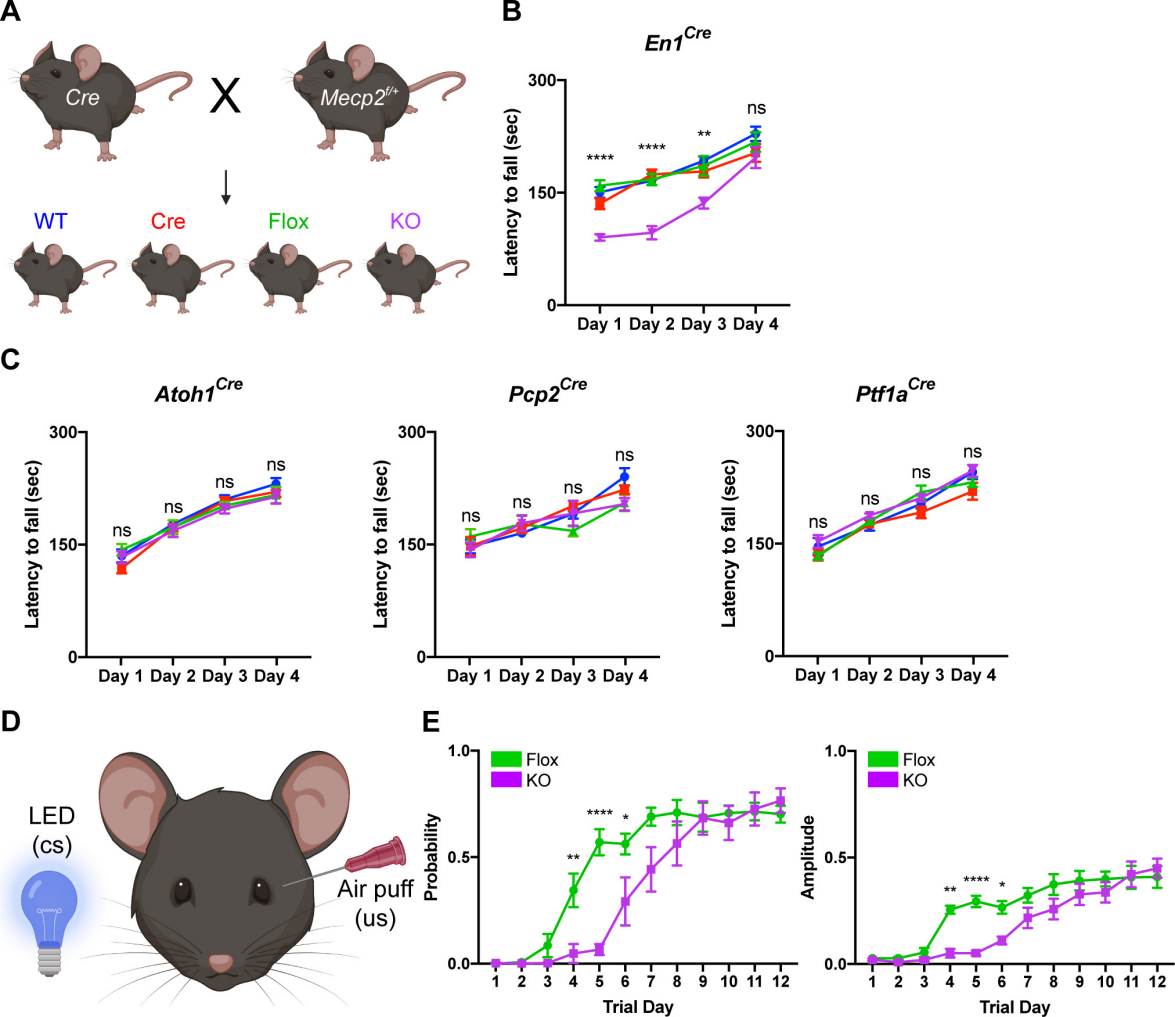
Deleting *Mecp2* from the cerebellum, but not its neuronal subtypes, causes motor learning deficits in 6-month-old mice. (**A**) Breeding scheme to generate WT, Cre, Flox, and KO mice. (**B**) Latency to fall on the rotarod over four training days in the *En1^Cre^* group. (**C**) Latency to fall on the rotarod over four training days in mice lacking *Mecp2* in the granule cells (*Atoh1^Cre^*), Purkinje cells (*Pcp2^Cr^*^e^), and Purkinje cells and molecular layer interneurons (*Ptf1a^Cre^*). (**D**) Schematic of eyeblink conditioning that pairs an LED light (conditioned stimulus, cs) with an air puff (unconditioned stimulus, us) to generate an anticipatory eyelid closure (conditioned response) before the air puff. (**E**) Response probability and amplitude of eyelid closure over 12 training days in Flox and KO mice. N = 8–17 biologically independent mice per group. Data are presented as mean ± s.e.m. Statistical significance was determined by two-way ANOVA with Tukey’s multiple comparisons test. ns (p>0.05), *(p<0.05), **(p<0.01), ****(p<0.0001). Figure 2—source data 1.Related to Figure 2.

Because the cerebellum is involved in motor coordination and learning (Mauk et al., 2000), we analyzed the motor performance of cerebellar KO mice using the rotarod (Deacon, 2013). In this assay, healthy mice spend progressively more time on a rotating rod as their motor skill improves, while mice with incoordination and motor learning impairments spend less time on the apparatus. Although the performance of 2- and 4-month-old cerebellar KO mice was normal compared to control mice, 6-month-old cerebellar KO mice had an initial motor learning delay that was overcome with additional training (Figure 2A,B; Figure 2—figure supplement 3A,B). These motor learning deficits were not due to abnormalities in general locomotor activity or strength (Figure 2—figure supplement 3C–F). Although the cerebellum is implicated in non-motor behaviors (Klein et al., 2016), we did not observe any deficits in sensorimotor gating, social behavior, or contextual fear memory in cerebellar KO mice (Figure 2—figure supplement 3G–K).

**Figure 2—figure supplement 1.**
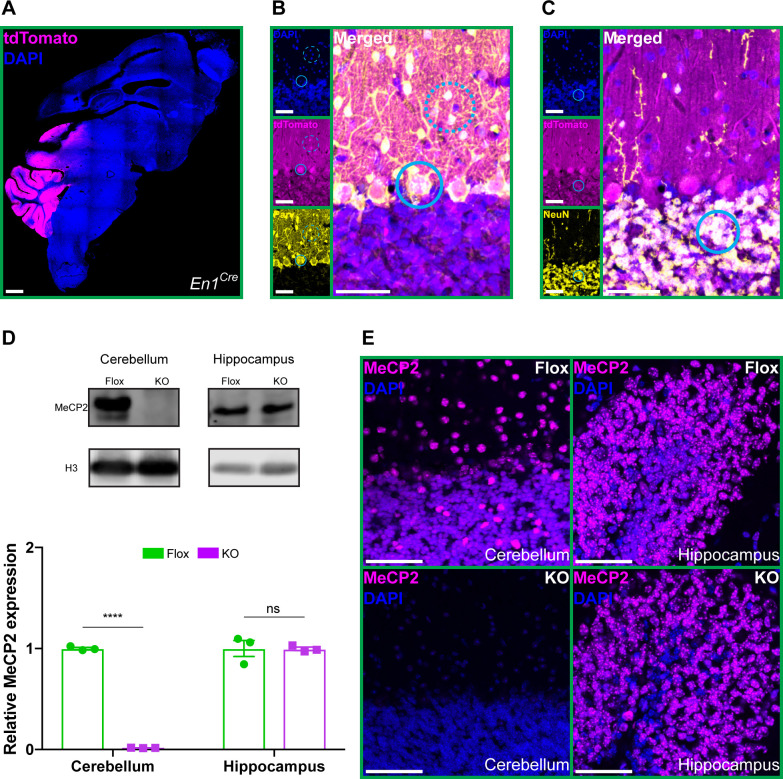
Deletion of *Mecp2* from the cerebellum. (**A**) *En1^Cre^* expression was determined by the pattern of tdTomato (magenta) in *En1^Cre^;Rosa26^lsl-tdTomato^* reporter mice. Scale bar, 1 mm. (**B**) Immunostaining showing co-expression of tdTomato (magenta) with Parvalbumin (yellow), a marker of Purkinje cells (solid cyan circle), and molecular layer interneurons (dashed cyan circle). Scale bar, 25 µm. (**C**) Immunostaining showing co-expression of tdTomato (magenta) with NeuN (yellow), a marker of granule cells (solid cyan circle). Scale bar, 25 µm. (**D**) Quantification of MeCP2 protein levels normalized to Histone H3 in the cerebellum and hippocampus of Flox and KO mice. (**E**) Immunostaining of MeCP2 (magenta) in the cerebellum and hippocampus of Flox and KO mice. Scale bar, 50 µm. N = 3 biologically independent mice per group. Data are presented as mean ± s.e.m. Statistical significance was determined by two-tailed, unpaired student’s t-test. ns (p>0.05), ****(p<0.0001). Figure 2—figure supplement 1—source data 1.Related to Figure 2—figure supplement 1.

**Figure 2—figure supplement 2.**
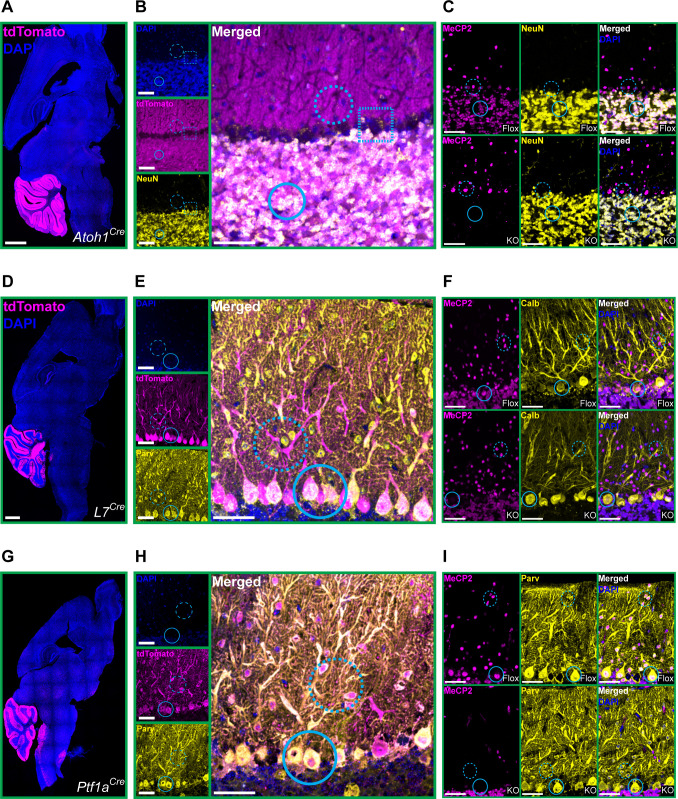
*Cre* expression and *Mecp2* deletion in cerebellar neuron subtypes. (**A**) *Atoh1^Cre^* expression was determined by the pattern of tdTomato (magenta) in *Atoh1^Cre^;Rosa26^lsl-tdTomato^* reporter mice. Scale bar, 1 mm. (**B**) Immunostaining in *Atoh1^Cre^;Rosa26^lsl-tdTomato^* reporter mice showing co-expression of tdTomato (magenta) with NeuN (yellow), a marker of granule cells (solid cyan circle), but not in cells of the Purkinje layer (dashed cyan square) or molecular layer (dashed cyan circle). Scale bar, 25 µm. (**C**) Immunostaining of MeCP2 (magenta) and NeuN (yellow) in the cerebellum showing the absence of MeCP2 in granule cells of KO mice (solid cyan circle), but not in cells of the Purkinje layer (dashed cyan circle). Scale bar, 25 µm. (**D**) *Pcp2^Cre^* expression was determined by the pattern of tdTomato (magenta) in *Pcp2^Cre^;Rosa26^lsl-tdTomato^* reporter mice. Scale bar, 1 mm. (**E**) Immunostaining in *Pcp2^Cre^;Rosa26^lsl-tdTomato^* reporter mice showing co-expression of tdTomato (magenta) with Parvalbumin (yellow) in the Purkinje cell layer (solid cyan circle), but not the molecular layer (dashed cyan circle). Scale bar, 25 µm. (**F**) Immunostaining of MeCP2 (magenta) and Calbindin (yellow) in the cerebellum showing the absence of MeCP2 in Purkinje cells of KO mice (solid cyan circle), but not in molecular layer interneurons (dashed cyan circle). Scale bar, 25 µm. (**G**) *Ptf1a^Cre^* expression was determined by the pattern of tdTomato (magenta) in *Ptf1a^Cre^;Rosa26^lsl-tdTomato^* reporter mice. Scale bar, 1 mm. (**H**) Immunostaining in *Ptf1a^Cre^;Rosa26^lsl-tdTomato^* reporter mice showing co-expression of tdTomato (magenta) with Parvalbumin (yellow) in the Purkinje layer (solid cyan circle) and molecular layer (dashed cyan circle). Scale bar, 25 µm. (**I**) Immunostaining of MeCP2 (magenta) and Parvalbumin (yellow) in the cerebellum showing the absence of MeCP2 in Purkinje cells (solid cyan circle) and molecular layer interneurons of KO mice (dashed cyan circle). Scale bar, 25 µm.

**Figure 2—figure supplement 3.**
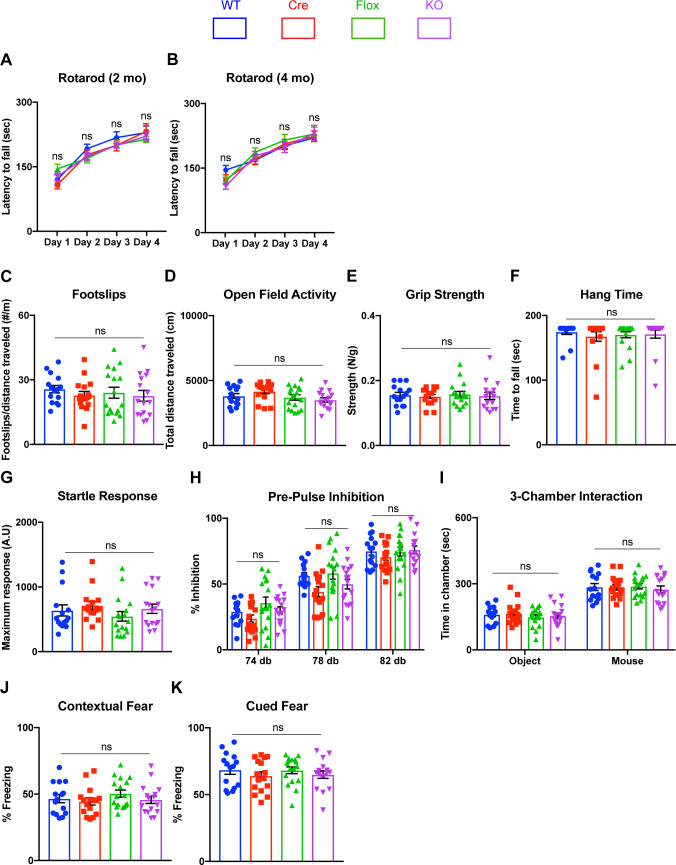
Deleting *Mecp2* from the cerebellum does not cause other behavioral abnormalities. (**A**) Latency to fall on the rotarod in 2-month-old mice. (**B**) Latency to fall on the rotarod in 4-month-old mice. A new cohort of WT, Cre, Flox, and KO mice was assessed for each time point. (**C**) Footslip count on the parallel rod assay normalized to total distance traveled. (**D**) Total distance traveled in the open-field assay. (**E**) Grip strength normalized to body weight. (**F**) Hang time on an inverted wire grid. (**G**) Maximum acoustic startle response to a 120 dB stimulus. (**H**) Pre-pulse inhibition to 74, 78, and 82 dB pre-pulses. (**I**) Interaction time in the three-chamber social interaction assay between a novel mouse or object. (**J**) Time spent freezing during contextual memory recall. (**K**) Time spent freezing during cued memory recall. N = 10–17 biologically independent mice per group. For (**C–K**), mice are 6 months old. Data are presented as mean ± s.e.m. Statistical significance was determined by one-way (**C–G, J–K**) or two-way ANOVA (**A–B, H–I**) with Tukey’s multiple comparisons test. ns (p>0.05). Figure 2—figure supplement 3—source data 1.Related to Figure 2—figure supplement 3.

Studies from other *Mecp2* KO mice have demonstrated that behavioral deficits originate from dysfunction in excitatory and inhibitory neurons (Chao et al., 2010; Meng et al., 2016). Furthermore, distinct behavioral phenotypes arise when *Mecp2* is removed from different subtypes of inhibitory neurons (Ito-Ishida et al., 2015; Mossner et al., 2020). For example, altered social behavior and seizures in *Mecp2* KO mice originate from dysfunction o parvalbumin- and somatostatin-expressing inhibitory neurons, respectively (Chao et al., 2010; Ito-Ishida et al., 2015). Therefore, we hypothesized that the motor learning deficits in cerebellar KO mice originate from the loss of *Mecp2* in excitatory granule cells, inhibitory Purkinje cells, and/or molecular layer interneurons. However, we did not detect rotarod deficits in cell type-specific KO animals at an age when cerebellar KO mice were symptomatic (Figure 2C).

In addition to the cerebellum, multiple brain regions including the cortex and basal ganglia contribute to motor learning on the rotarod (Sakayori et al., 2019). Therefore, we used eyeblink conditioning, an alternate task of motor learning for which the cerebellum is strictly necessary, to validate the motor learning deficits observed in cerebellar KO mice (Figure 2D; Heiney et al., 2014). Compared to control mice, cerebellar KO mice exhibited an initial delay in the probability and amplitude of eyelid closure that improved with additional training, suggesting that cerebellar-dependent motor learning is disrupted by the loss of *Mecp2* (Figure 2E). These results demonstrate that deleting *Mecp2* from the major cell types in the cerebellum causes a delay in motor learning.

### Purkinje cell firing is irregular in cerebellar KO mice

**Figure 3.**
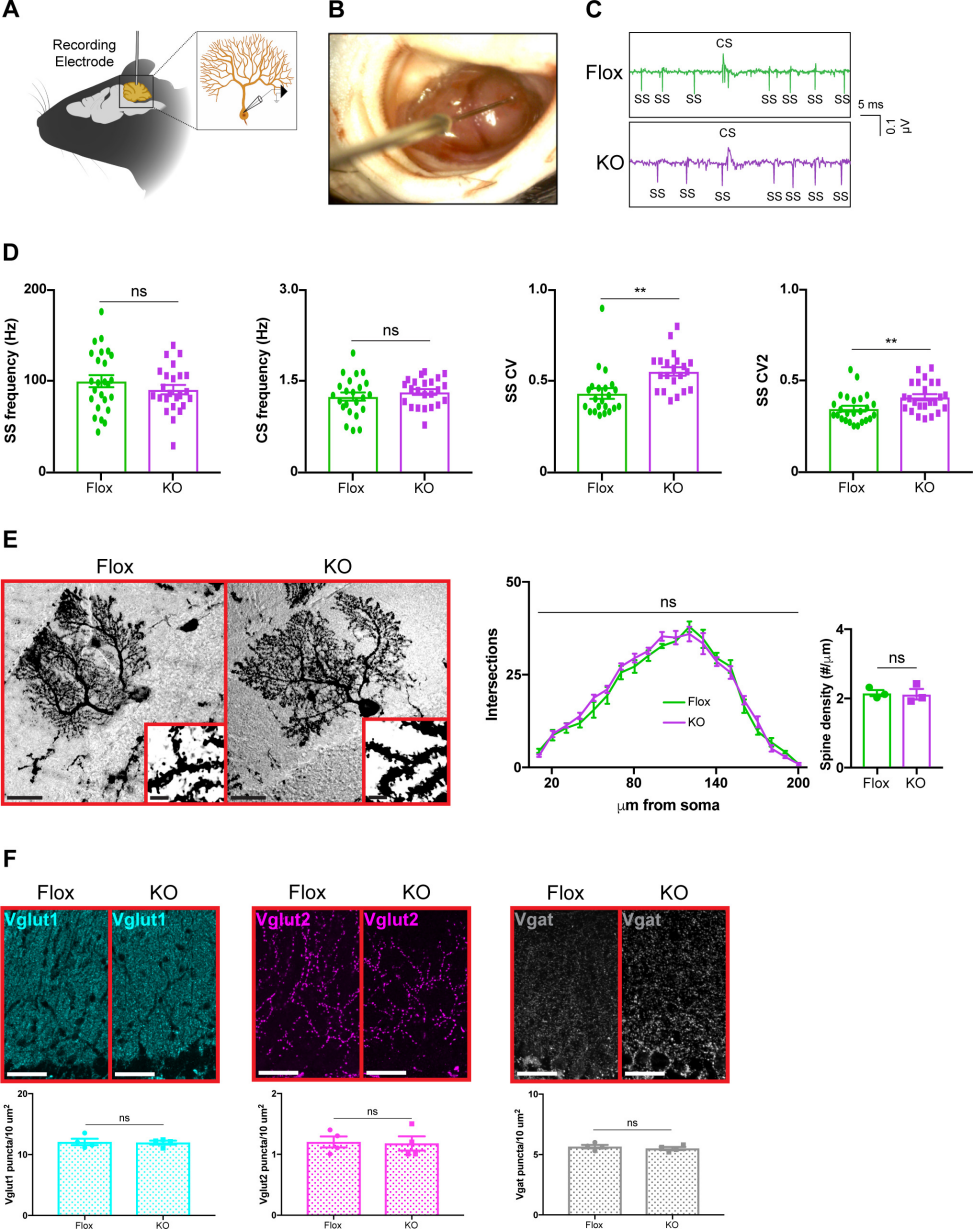
Purkinje cell firing rate is more irregular in cerebellar KO mice but is independent of overt morphological abnormalities. (**A**) Schematic of in vivo extracellular recording of Purkinje cells. (**B**) Photograph of a recording electrode inside a surgically implanted recording chamber. (**C**) Representative traces of Purkinje cell firing in Flox and KO mice displaying simple spikes (ss) and complex spikes (cs). (**D**) Simple spike firing rate, complex spike firing rate, coefficient of variation (CV), and coefficient of variation 2 (CV2). Simple and complex spikes were differentiated by their characteristic waveforms during offline analysis. (**E**) Golgi stain of Purkinje cells in Flox and KO mice. Scale bar, 25 µm. Inner panel demonstrates dendritic spines on Purkinje cells. Scale bar, 5 µm. Sholl analysis and spine density quantification in Flox and KO mice. (**F**) Staining and quantification of Vglut1 (cyan), Vglut2 (magenta), and Vgat (gray) puncta density in the cerebellum of Flox and KO mice. Scale bar, 25 µm. For (**D**), 23–27 neurons were analyzed from three biologically independent mice per group. For (**E**), 10–15 neurons were analyzed from three biologically independent mice per group. For (**F**), N = 4 biologically independent mice per group. Data are presented as mean ± s.e.m. Statistical significance was determined by two-tailed, unpaired student’s t-test (**D, F**) and two-way ANOVA with Tukey’s multiple comparisons test (**E**). ns (p>0.05), **(p<0.01). Figure 3—source data 1.Related to Figure 3.

Because removing *Mecp2* perturbs synaptic function in cortical and hippocampal neurons (Na et al., 2013; He et al., 2014; Meng et al., 2016; Ure et al., 2016), we hypothesized that alterations in the electrophysiological properties of cerebellar neurons might explain the motor phenotypes in cerebellar KO mice. To accomplish this, we monitored the activity of Purkinje cells in awake animals by recording their simple spikes, which originate from granule cell inputs, and complex spikes, which originate from inferior olivary neuron inputs (Schmolesky et al., 2002; Davie et al., 2008; Arancillo et al., 2015; Figure 3A–C). Because Purkinje cells are the final output stage of the cerebellar cortex, they serve as a reliable indicator of circuit dysfunction in the cerebellar circuit (Arancillo et al., 2015). We targeted Purkinje cells at the ventral portion of lobule V, targeting cells in the medial wall of the primary fissure, which includes the cerebellar microzone that supports eyeblink conditioning (Heiney et al., 2014; Ohmae and Medina, 2015). Focusing on lobule V instead of widely sampling from the cerebellum allowed us to compare the results of Purkinje cells within the same cerebellar subregion while minimizing the effect of zebrin-derived differences in firing proprieties because Purkinje cells in lobule V are largely zebrin negative (Ozol et al., 1999; Zhou et al., 2014; Peter et al., 2016). Although the mean firing rates were unchanged, simple spike firing was more irregular in cerebellar KO mice, as was evident by an increase in the coefficient of variation (CV) and coefficient of variation 2 (CV2) (Figure 3D). This electrophysiological abnormality was not due to defects in Purkinje cell morphology or the density of excitatory and inhibitory synaptic puncta (Figure 3E,F). Thus, deleting *Mecp2* from the cerebellum disrupts aspects of Purkinje cell firing without overt neuroanatomical abnormalities.

### H3K9me3 levels in heterochromatic foci are reduced in cerebellar KO mice

**Figure 4.**
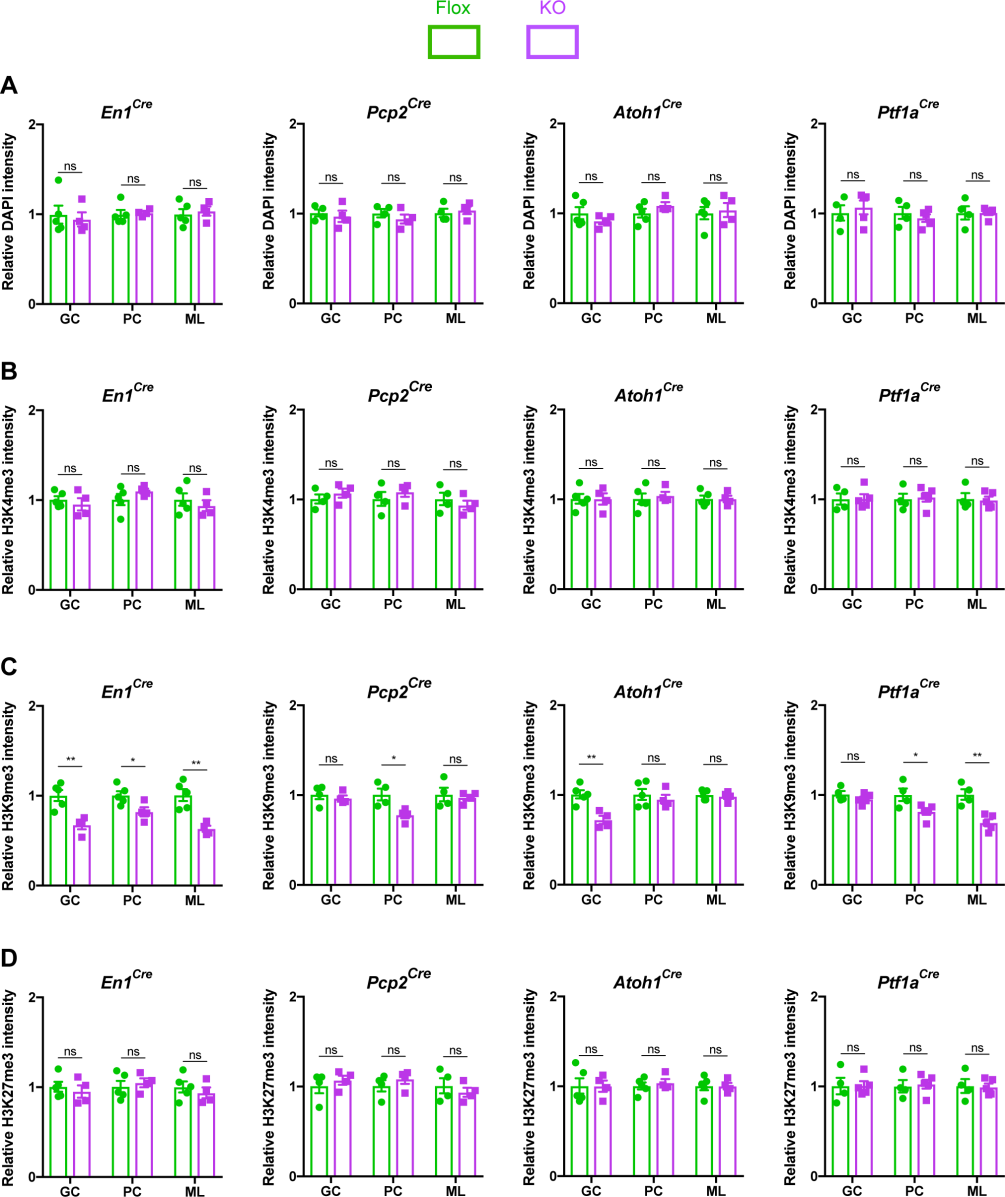
The loss of *Mecp2* in cerebellar neurons disrupts histone methylation in heterochromatic foci. The intensity of DAPI and histone methylation marks was measured in the heterochromatic foci of granule cells (GC), Purkinje cells (PC), and molecular layer interneurons (ML) in Flox and KO mice. (**A**) Normalized DAPI intensity in heterochromatic foci. (**B**) Normalized H3K4me3 intensity in heterochromatic foci. (**C**) Normalized H3K9me3 intensity in heterochromatic foci. (**D**) Normalized H3K27me3 intensity in heterochromatic foci. 15–20 neurons were analyzed per mouse. Data were normalized to the values of Flox mice. N = 4–5 biologically independent mice per group. Data are presented as mean ± s.e.m. Statistical significance was determined by two-tailed, unpaired student’s t-test. ns (p>0.05), *(p<0.05), **(p<0.01). Figure 4—source data 1.Related to Figure 4.

MeCP2 is a nuclear protein that binds methylated cytosines on DNA throughout the genome and regulates gene expression (Tillotson and Bird, 2019). Multiple studies have demonstrated that MeCP2 regulates heterochromatin structure in cortical and hippocampal neurons (Baker et al., 2013; Linhoff et al., 2015; Ito-Ishida et al., 2020). We hypothesized that cerebellar neurons in KO mice would display similar structural abnormalities. To test this, we assayed the intensity of DAPI, H3K4me3, H3K9me3, and H3K27me3 in the heterochromatic foci of granule cells, Purkinje cells, and molecular layer interneurons (Figure 4—figure supplement 1A–C). We analyzed mice lacking *Mecp2* in the cerebellum (*En1^Cre^*) as well as in its neuronal subtypes (*Atoh1^Cre^*, *Pcp2^Cre^*, and *Ptf1a^Cre^*) and compared each KO strain to their control littermates. To avoid contaminating our results with measurements from glia, which have different levels of histone methylation than neurons (Girdhar et al., 2018), we only analyzed cells that expressed NeuN, which labels granule cells, and RORα, which labels Purkinje cells and molecular layer interneurons (Figure 4—figure supplement 2A–C). In cerebellar KO mice (*En1^Cre^*), the level of H3K9me3 was reduced in the heterochromatic foci of granule cells, Purkinje cells, and molecular layer interneurons, but the levels of DAPI, H3K4me3, and H3K27me3 were unaffected (Figure 4A–D). Interestingly, this phenomenon was also observed in granule cells of *Atoh1^Cre^* KO mice, Purkinje cells of *Pcp2^Cre^* KO mice, and Purkinje cells and molecular layer interneurons of *Ptf1a^Cre^* KO mice (Figure 4A–D). Thus, deleting *Mecp2* from the cerebellum reduces levels of H3K9me3 in heterochromatic foci, indicating that some aspects of heterochromatin architecture are altered by the loss of *Mecp2*. It is noteworthy that this change was present in mice lacking *Mecp2* in neuronal subtypes of the cerebellum, even though these mice lacked behavioral phenotypes (Figure 2C).

**Figure 4—figure supplement 1.**
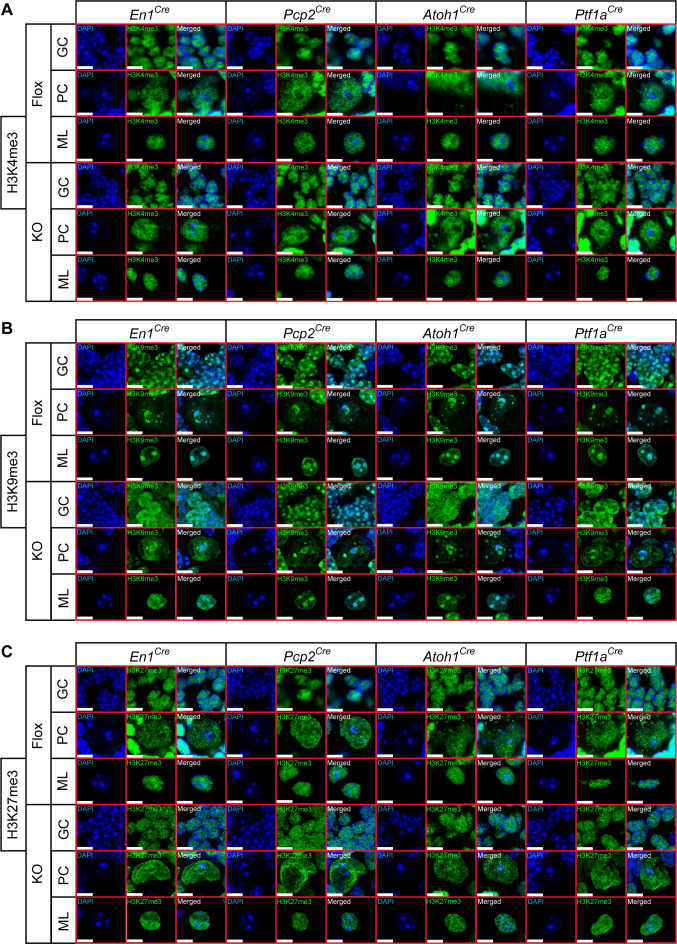
Heterochromatin architecture in mice lacking *Mecp2* in cerebellar neurons. (**A**) Representative images of cerebellar neurons in Flox and KO mice showing DAPI and H3K4me3. Scale bar, 5 µm. (**B**) Representative images of cerebellar neurons in Flox and KO mice showing DAPI and H3K9me3. Scale bar, 5 µm. (**C**) Representative images of cerebellar neurons in Flox and KO mice showing DAPI and H3K27me3. Scale bar, 5 µm.

**Figure 4—figure supplement 2.**
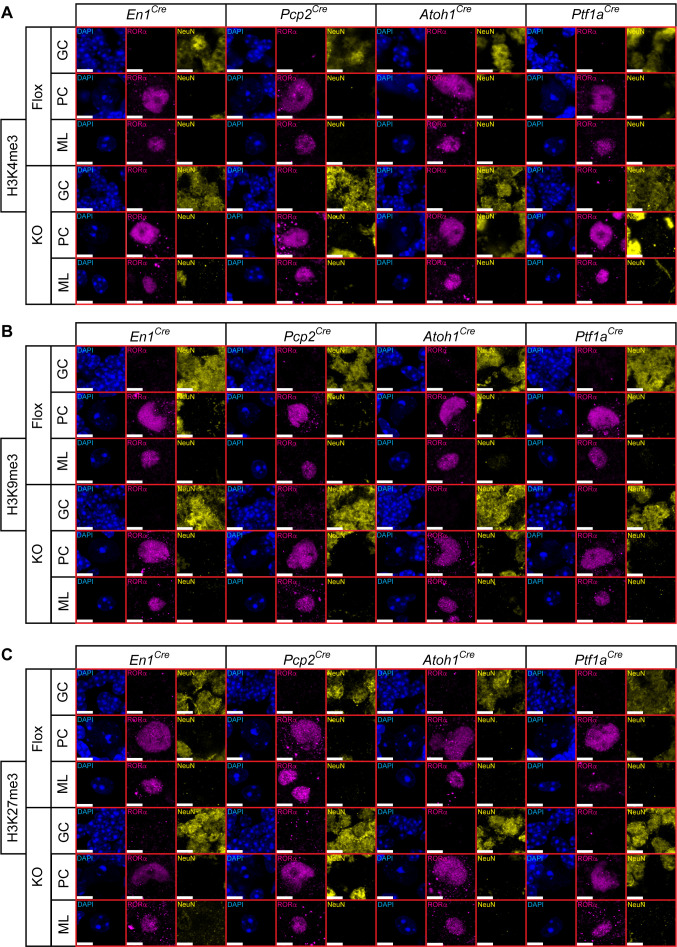
Cerebellar neurons were identified by the expression of RORα and NeuN. (**A**) Neurons stained for H3K4me3 from Figure 4—figure supplement 1 were co-stained for RORα and NeuN to identify molecular layer interneurons (RORα), Purkinje cells (RORα), and granule cells (NeuN). Scale bar, 5 µm. (**B**) Neurons stained for H3K9me3 from Figure 4—figure supplement 1 were co-stained for RORα and NeuN to identify molecular layer interneurons (RORα), Purkinje cells (RORα), and granule cells (NeuN). Scale bar, 5 µm. (**C**) Neurons stained for H3K27me3 from Figure 4—figure supplement 1 were co-stained for RORα and NeuN to identify molecular layer interneurons (RORα), Purkinje cells (RORα), and granule cells (NeuN). Scale bar, 5 µm.

## Discussion

Our study revealed three important features of cerebellar dysfunction that occur following the loss of *Mecp2*. First, we did not observe non-motor phenotypes in cerebellar KO mice even though the cerebellum is implicated in non-motor behaviors such as social interaction and cognition (Mauk et al., 2000; Tsai et al., 2012; Klein et al., 2016). This suggests that non-motor phenotypes in Rett syndrome are likely not caused by cerebellar dysfunction. Second, we did not observe motor deficits in any of the cell type-specific KO mice. The same phenomenon is seen for the sensorimotor gating deficits of *Mecp2* null mice, which are present in mice lacking *Mecp2* in all inhibitory neurons but not in individual subtypes of inhibitory neurons (Chao et al., 2010; Ito-Ishida et al., 2015). This suggests that the cerebellar-related motor deficits are the result of combined dysfunction in the entire circuit rather than dysfunction in a single cell type. Finally, the behavioral deficits in cerebellar KO mice were milder than mice lacking *Mecp2* in the cortex and basal ganglia. In cerebellar KO mice, the phenotypes were restricted to motor learning and appeared in 6-month-old mice, whereas the motor deficits in mice lacking *Mecp2* in the cortex and basal ganglia are more profound and arise in 2-month-old mice (Chen et al., 2001; Gemelli et al., 2006; Su et al., 2015). Thus, the motor symptoms of Rett syndrome arise from a combination of cerebellar, cortical, and basal ganglia dysfunction.

Purkinje cells serve as the final output stage of the cerebellar cortex and integrate synaptic inputs from granule cells, molecular layer interneurons, and inferior olivary neurons (Arancillo et al., 2015). Thus, they are an ideal cell type for determining if functional defects occurred at any point in the cerebellar circuit. The irregular firing rates of Purkinje cells suggest that the motor phenotypes of KO mice are caused by defects in the cerebellar circuitry. Although the synaptic changes in cerebellar KO mice were mild compared to those observed in other *Mecp2* KO models (Chao et al., 2010; Meng et al., 2016), they may still contribute to the behavioral deficits since similar findings are observed in other mouse models of cerebellar dysfunction. For example, the loss of the α3 isoform of the Na^+^/K^+^ pump in mice causes motor incoordination and dystonia (Calderon et al., 2011). In these mice, the mean firing rate of their Purkinje cells is normal, but the firing rates are irregular, similar to what we observed in cerebellar KO mice (Fremont et al., 2014). Just like cerebellar KO mice, the Purkinje cells of *Car8^wdl^* mice have irregular simple spike activity, which ultimately contributes to motor incoordination on the rotarod (White et al., 2016). Because Purkinje cells integrate input from multiple cell types, the irregularity of simple spike firing could arise from a combination of factors including impairments in intrinsic Purkinje cell responses, abnormal excitatory input from granule cells, and/or perturbations in synaptic modulation from molecular layer interneurons.

The behavioral and synaptic impairments were accompanied by a reduction in the level of H3K9me3 in the heterochromatic foci of cerebellar neurons. This defect was present in a cell-autonomous manner when *Mecp2* was removed from granule cells, Purkinje cells, and molecular layer interneurons (*En1^Cre^*) and when *Mecp2* was removed from subtypes of cerebellar neurons (*Atoh1^Cre^*, *Pcp2^Cre^*, and *Ptf1a^Cre^*). Yet, these mice lacked behavioral phenotypes. Similar to our findings, changes in the heterochromatic foci of *Mecp2*-null hippocampal neurons are present in presymptomatic *Mecp2^+/–^* female mice (Ito-Ishida et al., 2020). In symptomatic *Mecp2^+/−^* female mice, the levels of H3K4me3 and H3K27me3, but not H3K9me3, were elevated in *Mecp2*-null hippocampal neurons (Ito-Ishida et al., 2020). In addition, H4K20me3 is abnormally distributed in *Mecp2*-null hippocampal neurons, but not cerebellar granule neurons (Linhoff et al., 2015). Thus, our results indicate that alterations in heterochromatin architecture do not always coincide with behavioral abnormalities and are influenced by the particular cell type and histone modification.

The *Cre-LoxP* system allowed us to selectively remove *Mecp2* from various cerebellar cell types, but for some of these mouse strains, *Cre* is expressed outside the cerebellum. *En1^Cre^* is expressed in the midbrain, spinal cord interneurons, and muscle (Atit et al., 2006; Joyner and Zervas, 2006; Bikoff et al., 2016). Fortunately, we do not believe that removing *Mecp2* from these regions confounded our behavioral observations. First, removing *Mecp2* from dopaminergic neurons, including those in the midbrain, does not affect rotarod performance (Samaco et al., 2009). Second, although the loss of *Mecp2* in spinal cord interneurons could contribute to defects on the rotarod, it is unlikely to affect eyeblink conditioning as this reflex is mediated by circuits in the cerebellum and brainstem (Bracha, 2004). Third, the loss of *Mecp2* in skeletal muscle does not affect muscle morphology or physiology (Conti et al., 2015). Finally, although *Ptf1a^Cre^* removes *Mecp2* from Purkinje cells and molecular layer interneurons, the absence of motor deficits in *Pcp2^Cre^* KO mice, which only targets Purkinje cells, indicates that the loss of *Mecp2* from molecular layer interneurons does not cause the behavioral deficits seen in *En1^Cre^* KO mice. Moreover, *Ptf1a^Cre^* is expressed in inferior olivary neurons, a main source of input to the cerebellum (Hoshino et al., 2005). The absence of motor phenotypes in *Ptf1a^Cre^* KO mice suggests that the loss of *Mecp2* in inferior olivary neurons does not disrupt motor function in mice.

A unique and interesting finding was the improvement in motor learning after additional training in cerebellar KO mice. To our knowledge, this phenomenon is not observed in other *Mecp2* KO mice (Li and Pozzo-Miller, 2012; Lombardi et al., 2015). However, a related effect is seen in female *Mecp2* heterozygous mice in which their memory deficits are rescued with forniceal deep brain stimulation (DBS) (Hao et al., 2015; Lu et al., 2016). The effects of DBS on brain circuitry share similarities to that of repetitive activation during training (De Zeeuw and Ten Brinke, 2015; Herrington et al., 2016; Lu et al., 2016; Langille and Brown, 2018). In cerebellar KO mice, it is possible that activation of the cerebellar circuitry during training improves their motor phenotypes by enhancing synaptic function in a manner similar to the proposed mechanism of DBS. This also raises the possibility that repetitive circuit activation via training could improve other behavioral deficits in *Mecp2* KO mice.

Taken together, our results reveal that cerebellar dysfunction contributes to motor deficits following the loss of *Mecp2*. Interestingly, the behavioral, synaptic, and cellular deficits in cerebellar KO mice were relatively mild compared to other *Mecp2* KO models. So, even though *Mecp2* is broadly expressed throughout the brain, neuronal subtypes between brain regions, and even those within a brain region, respond differently to the loss of *Mecp2*. As future studies seek to better define the function of *Mecp2* and use this knowledge to design effective therapies, it is important to keep in mind that the functional consequences of *Mecp2* loss are context specific.

## Materials and methods

**Key resources table:** 

Reagent type (species) or resource	Designation	Source or reference	Identifiers	Additional information
Antibody	Rabbit polyclonal anti-Histone H3	Abcam	RRID:AB_302613 Cat# ab1791	1:20,000
Antibody	Rabbit monoclonal anti-MeCP2	Cell Signaling Technologies	RRID:AB_2143849 Cat# 3456	1:1000
Antibody	Mouse monoclonal anti-MeCP2	Abcam	RRID:AB_881466 Cat# ab50005	1:500
Antibody	Mouse monoclonal anti-NeuN	Millipore Sigma	RRID:AB_2298772 Cat# MAB377	1:250
Antibody	Mouse monoclonal anti-Calbinin-D28K	Swant	RRID:AB_10000347 Cat# 300	1:10,000
Antibody	Rabbit polyclonal anti-Parvalbumin	Swant	RRID:AB_2631173 Cat# PV27	1:1000
Antibody	Rabbit polyclonal anti-Vglut1	Synaptic Systems	RRID:AB_887877 Cat# 135 302	1:1000
Antibody	Guinea pig polyclonal anti-Vglut2	Synaptic Systems	RRID:AB_887884 Cat# 135 404	1:1000
Antibody	Guinea pig polyclonal anti-Vgat	Synaptic Systems	RRID:AB_1106810 Cat# 131 005	1:1000
Antibody	Rabbit polyclonal anti-histone H3 (tri methyl K4)	Cell Signaling Technologies	RRID:AB_2616028 Cat# 9751	1:500
Antibody	Rabbit polyclonal anti-histone H3 (tri methyl K9)	Abcam	RRID:AB_306848 Cat# ab8898	1:500
Antibody	Rabbit polyclonal anti-histone H3 (tri methyl K27)	Millipore Sigma	RRID:AB_310624 Cat# 07–449	1:500
Antibody	Goat polyclonal anti-RORα	Santa Cruz Biotechnology	RRID:AB_655755 Cat# sc-6062	1:250
Antibody	Goat anti-mouse IgG Alexa Fluor 488	Thermo Fischer	RRID:AB_2534069 Cat# A-11001	1:500
Antibody	Goat anti-guinea pig IgG Alexa Fluor 555	Thermo Fischer	RRID:AB_2535856 Cat# A-21435	1:500
Antibody	Goat anti-rabbit IgG Alexa Fluor 647	Thermo Fischer	RRID:AB_2535812 Cat# A-21244	1:500
Antibody	Donkey anti-rabbit IgG Alexa Fluor 488	Thermo Fischer	RRID:AB_2535792 Cat# A-21206	1:500
Antibody	Donkey anti-goat IgG Alexa Fluor 555	Thermo Fischer	RRID:AB_2535853 Cat# A-21432	1:500
Antibody	Donkey anti-mouse IgG Alexa Fluor 647	Thermo Fischer	RRID:AB_162542 Cat# A-31571	1:500
Commercial assay, kit	Paraformaldehyde	Millipore Sigma	Cat# 158127	
Commercial assay, kit	Pierce BCA Protein Assay	Thermo Fischer	Cat# 23225	
Commercial assay, kit	FD Rapid Golgi Stain Kit	FD Neurotechnologies	Cat# PK401	
Strain, strain background (*Mus musculus*)	(C57BL/6J) *Rosa26^lsl-tdTomato^*	The Jackson Laboratory	RRID: IMSR_JAX:007914	
Strain, strain background (*Mus musculus*)	(C57BL/6J) *En1^Cre^*	The Jackson Laboratory	RRID: IMSR_JAX:007916	
Strain, strain background (*Mus musculus*)	(C57BL/6J) *Atoh1^Cre^*	The Jackson Laboratory	RRID:IMSR_JAX:011104	
Strain, strain background (*Mus musculus*)	(C57BL/6J) *Pcp2^Cre^*	The Jackson Laboratory	RRID:IMSR_JAX:004146	
Strain, strain background (*Mus musculus*)	(C57BL/6J) *Ptf1a^Cre^*	The Jackson Laboratory	RRID:IMSR_JAX:007909	
Strain, strain background (*Mus musculus*)	(C57BL/6J) *Mecp2^flox/+^* and *Mecp2^flox/flox^*	The Jackson Laboratory	RRID:IMSR_JAX:007177	
Other	DAPI stain	Thermo Fischer	RRID:AB_2629482 Cat# D-1306	
Other	Tissue-Tek Optimum Cutting Temperature Compound	Sakura	Cat# 4583	
Other	Superfrost Plus microscope slides	Thermo Fischer	Cat# 12-550-15	
Other	ProLong Gold Antifade mounting medium	Thermo Fischer	Cat# P10144	
Other	NuPAGE LDS sample buffer	Thermo Fischer	Cat# NP0007	
Other	NuPAGE Sample reducing agent	Thermo Fischer	Cat# NP0004	
Other	15-well NuPAGE 4–12% Bis–Tris Gel	Thermo Fischer	Cat# NP0336BOX	
Other	15-well NuPAGE 4–12% Bis–Tris Gel	Thermo Fischer	Cat# NP0336BOX	
Other	PVDF blotting membrane	GE Healthcare Life Sciences	Cat# 10600021	
Other	Odyssey TBS Blocking Buffer	LI-COR Biosciences	Cat# 927–50000	
Software, algorithm	Spike2	Cambridge Electronic Design	RRID:SCR_000903	
Software, algorithm	MATLAB	Mathworks	RRID: SCR_001622	
Software, algorithm	Image Studio Lite	LI-COR Biosciences	RRID:SCR_013715	
Software, algorithm	ImageJ-Fiji	Other	RRID:SCR_002285	
Software, algorithm	Neurolucida 360	MBF Biosciences	RRID:SCR_016788	
Software, algorithm	Neurolucida Explorer	MBF Biosciences	RRID:SCR_017348	
Software, algorithm	Imaris	Bitplane	RRID:SCR_007370	
Software, algorithm	Prism	GraphPad Software	RRID: SCR_002798	

### Animals

Mice were maintained on a C57B/6J background on a 14 hr light: 10 hr dark cycle with standard mouse chow and water ad libitum. Mice were group housed up to five mice per cage. All behavioral experiments were performed during the light cycle at the same time of day. The following Cre-expressing mice were used for breeding: *En1^Cre^* (*En1^tm2(cre)Wrst^*/J), *Atoh1^Cre^* (B6.Cg-Tg(Atoh1Cre)1Bfri/J), *Pcp2^Cre^* (B6.129-Tg(Pcp2-cre)2Mpin/J), and *Ptf1a^Cre^* (*Ptf1a^tm1(cre)Hnak^*/RschJ). Cre-expressing male mice were bred to *Mecp2^flox/+^* female mice (Chen et al., 2001) to generate WT, Cre, Flox, and KO mice for behavior experiments. Cre-expressing male mice were bred to *Mecp2^flox/flox^* female mice (Chen et al., 2001) to generate Flox and KO mice for eyeblink conditioning, histological, and electrophysiological experiments. Mice were obtained from the Jackson Laboratories and maintained by breeding mice to wild-type C57B/6J mice. Only male offspring were used because the mosaic nature of *Mecp2* expression in females would confound the results (Calfa et al., 2011). Behavioral, histological, and electrophysiological analyses were performed blind to genotypes. The Baylor College of Medicine Institutional Animal Care and Use Committee approved all research and animal care procedures.

### Behavioral assays

For each test, mice were habituated in the room for 30 min. A light intensity of 150 lx and 60 dB background white noise was presented during habituation and testing. All assays were performed at the same time of day.

### Rotarod

Mice were placed on an accelerating rotarod apparatus (Ugo Basile), while the cylinder increased from 5 rpm to 40 rpm over a 5 min period. Latency to fall was measured when the mouse fell off the apparatus or rode the cylinder for two consecutive revolutions without regaining control. Mice were tested over 4 days, with each day consisting of four attempts and a 30 min rest after each attempt.

### Eyeblink conditioning

Eyeblink conditioning was performed as previously described (Heiney et al., 2014). Briefly, animals were anesthetized with isoflurane (1.5–2% by volume in O_2_, SuriVet). A midline incision was made to expose the skull, and two small screws were placed on either side of the midline at bregma. A thin stainless steel headplate was placed on the skull such that the screws fit in the hole in the headplate. The plate was adhered to the skull using Metabond cement (Parkell). After 5 days of recovery, mice were habituated to the head restraint in the testing chamber for 2 days prior to training for 1 hr. Each training session consisted of 100 trials of the conditioned stimulus (CS, blue LED light) paired with the unconditioned stimulus (US, 20–30 psi periocular air puff). The interstimulus interval was 200 ms with an intertrial interval of at least 10 s. The pressure of the periocular air puff was set for each mouse to elicit a full reflexive blink. The conditioned response (CR, eyelid closure) was monitored using infrared illumination and a high-speed camera (Allied Vision) combined with MATLAB (Mathworks) using a custom-written software and acquisition toolbox. Mice were trained for 12 days after habituation. Eyelid traces were normalized to the full blink range. Trials were considered to contain a CR if the eyelid closure exceeded 5% from the baseline mean within the CS–US interval. The CR probability was quantified as the number of CRs divided by the total number of trials for each day. The CR amplitude was quantified as the maximum eyelid position within the CS-US interval relative to the trial baseline position for each day.

### Open-field assay

Mice were placed in a clear, open Plexiglas box (40 × 40 × 30 cm, Stoelting) with an overhead camera and photo beams to record horizontal and vertical movements. Activity was measured over 10 min and quantified using ANY-maze (Stoelting).

### Parallel rod footslip

Mice were placed into the center of a wire grid laid in an open-field chamber (Accuscan) for 10 min. The number of footslips through the wire grid was recorded and analyzed using ANY-maze (Stoelting). The number of footslips was normalized to the total distance traveled.

### Grip strength

Mice were held by the tail and allowed to grasp the bar of a grip strength meter (Chatillon-Ametek) with both forepaws. The mouse was pulled away from the bar until it released from the bar. The maximum force generated was averaged over three trials and normalized to the weight of the mouse.

### Wire hang time

Mice were allowed to grasp the middle of a 3 mm plastic coated wire suspended six inches above a plastic-covered foam pad. The plastic wire was inverted for a maximum of 180 s or until the mouse fell off.

### Three-chamber interaction

During the habituation phase, mice were placed in the middle of the three-chamber apparatus (Ugo Basile) containing two empty barred cages in the right and left chambers for 10 min. During the social interaction phase, an age-matched C57BL/6J wild-type male mouse was placed in one cage and a black Lego block of similar size was placed in the other. Partner mice were habituated to the chamber for 1 hr per day for two consecutive days before testing. The test mouse was returned to the middle zone and allowed to explore the chamber for 10 min. Mouse movement was recorded and analyzed using ANY-maze (Stoelting).

### Fear-conditioning assay

On the first day, mice were placed in a holding room and delivered to the testing room in a temporary cage. Mice were trained in a fear-conditioning chamber (Med Associates, Inc) that delivers an electric shock paired with a tone. This device was located inside a soundproof box that contained a digital camera and loudspeaker. Each mouse was placed individually in the chamber and left undisturbed for 2 min. A tone (80 dB, 5 kHz, 30 s) coincided with a foot-shock (2 s, 0.7 mA) and was repeated after 1 min. The apparatus was cleaned with isopropanol. The mouse was returned to the temporary cage after an additional minute and returned the home-cage in the holding room. Fear memory was assessed after 1 day of training. To test contextual fear memory, mice were placed in the original environment without a tone or foot-shock for 5 min. Mice were returned to their home-cage in the holding room. To test cued fear memory, mice were returned to the testing room and placed in the chamber, which was modified to distinguish it from the original context. The chamber was made triangular with the addition of white panels, cleaned with 70% ethanol, and scented with a cup of vanilla extract under the floor. The mouse was allowed to explore the novel environment for 3 min, after which the original tone (80 dB, 5 kHz, 3 min) was presented. Mouse movement was recorded and analyzed using ANY-maze (Stoelting). Freezing was scored only if the animal was immobile for at least 1 s.

### Acoustic startle and pre-pulse inhibition

Mice were placed in a Plexiglas tube and allowed to habituate for 5 min with a 70 dB background noise. The test sessions consisted of six trials of each sound stimuli lasting 20 ms: no stimulus, a 120 dB sound burst, or a 120 dB sound burst with a 74 dB, 78 dB, or 82 dB pre-pulse stimuli presented 100 ms before the startle stimulus. The maximum startle response was recorded and analyzed during the 65 ms period following the onset of the startle stimulus (SR-Lab). Pre-pulse inhibition was calculated as 1 − (startle response with pre-pulse stimulus/startle response only × 100).

### Histology and immunofluorescence staining

For immunofluorescence, animals were transcardially perfused with 50 ml ice-cold 4% paraformaldehyde in 0.1 M phosphate-buffered saline (PBS). Brains were dissected, post-fixed overnight at 4°C, washed with 0.1 M PBS, and placed in 30% sucrose in 0.1 M PBS for 24 hr. Brains were embedded in Tissue-Tek Optimum Cutting Temperature Compound (Sakura) and stored at −80°C until further use. Fifty micrometer floating sections were cut using a cryostat (Leica) and collected in 0.1 M PBS. Sections were incubated in blocking solution (0.3% Triton X-100, 5% normal goat serum, or 5% normal donkey serum in 0.1 M PBS) for 1 hr at room temperature followed by primary antibody in blocking solution for 24 hr at 4°C. The following primary antibodies were used: rabbit anti-MeCP2 (1:1000, Cell Signaling Technology), mouse anti-MeCP2 (1:500, Abcam), mouse anti-NeuN (1:250, Millipore Sigma), mouse anti-Calbinin-D28K (1:10,000, Swant), rabbit anti-parvalbumin (1:1000, Swant), rabbit anti-Vglut1 (1:1000, Synaptic Systems), guinea pig anti-Vglut2 (1:1000, Synaptic Systems), guinea pig anti-Vgat (1:1000, Synaptic Systems), rabbit anti-Histone H3 (tri methyl K4) (1:500, Cell Signaling Technology), rabbit anti-Histone H3 (tri methyl K9) (1:500, Abcam), rabbit anti-Histone H3 (tri methyl K27) (1:500, Millipore Sigma), and goat anti-RORα (1:250, Santa Cruz Biotechnology). Sections were washed with 0.1 M PBS and incubated in secondary antibody for 2 hr at room temperature. The following secondary antibodies were used: goat anti-mouse IgG Alexa Fluor 488 (1:500, Thermo Fisher), goat anti-guinea pig IgG Alexa Fluor 555 (1:500, Thermo Fisher), goat anti-rabbit IgG Alexa Fluor 647 (1:500, Thermo Fisher), donkey anti-rabbit IgG Alexa Fluor 488 (1:500, Thermo Fisher), donkey anti-goat IgG Alexa Fluor 555 (1:500, Thermo Fisher), and donkey anti-mouse IgG Alexa Fluor 647 (1:500, Thermo Fisher). Sections were washed with 0.1 M PBS, counterstained with 1 mM DAPI (Thermo Fisher) for 5 min, and mounted on electrostatic Superfrost Plus microscope slides (Thermo Fisher) with ProLong Gold Antifade mounting medium (Thermo Fisher). Slides were cured overnight at room temperature and stored at 4°C prior to imaging. For Golgi–Cox staining, the brains were removed from the skull and processed using the FD Rapid Golgi Stain Kit (FD Neurotechnologies). All steps were carried out according to the manufacturer’s instructions. The tissue was sectioned at 100 μm and transferred to electrostatic glass slides and mounted with ProLong Gold Antifade mounting medium (Thermo Fisher). For Nissl staining, 50 μm frozen sections were mounted onto electrostatic Superfrost Plus microscope slides (Thermo Fisher), dehydrated overnight in 1:1 ethanol:chlorofom, rehydrated in 100% and 95% ethanol, stained with 0.1% cresyl violet, washed in 95% and 100% ethanol, cleared with xylene, and mounted with ProLong Gold Antifade mounting medium (Thermo Fisher). Slides were stored at room temperature prior to imaging.

### Imagining and quantification

Brightfield images were captured with the AxioCam MRc5 camera (Zeiss) mounted on an Axio Imager (Zeiss). Confocal images were captured with the TCS SP8 microscope (Leica) using a 10× or 63× objective. Z-stack images were acquired at 10 μm steps (tdTomato reporter characterization), 0.5 μm steps (tdTomato reporter characterization, MeCP2 characterization, and morphological analysis), and 0.1 μm steps with an additional 10× zoom (heterochromatin analysis). Laser settings were set above background levels based on the signal intensity of tissue stained only with the secondary antibody and kept consistent across samples in each experiment. MeCP2-expressing cells were counted using the Coloc2 function in ImageJ-Fiji (Schindelin et al., 2012). Vglut1, Vglut2, and Vgat puncta were counted using the Analyze Particles function in ImageJ-Fiji (Schindelin et al., 2012). Neuronal structure reconstruction, Sholl analysis, and spine quantification were performed with Neurolucida 360 and Neurolucida Explorer (MBF Bioscience). Individual nuclei were isolated, and heterochromatic foci were visualized using the surface tool in Imaris (Bitplane). The relative signal intensity was calculated by dividing the signal intensity in the heterochromatic foci by the total signal intensity in the nucleus. The values for KO mice were normalized to the values for Flox mice.

### Purkinje cell electrophysiology

Single-unit extracellular recording was performed as previously described (Heiney et al., 2018). A 2–3 mm diameter craniotomy was opened over the right side of the cerebellum (6.5 mm posterior and 2.0 mm lateral from bregma), and the dura was protected by a layer of Kwik-Sil (WPI). A custom 3D printed recording chamber and interlocking lid (NeuroNexus) was secured over the craniotomy with dental acrylic to provide additional protection. After 5 days of recovery, the mouse was fixed in place on a treadmill via a previously implanted headplate. Purkinje cell simple spikes (SSpk) and complex spikes (CSpk) were isolated using a tetrode (Thomas Recording, AN000968) acutely driven into the cerebellar cortex with microdrives mounted on a stereotactic frame (Narishige MMO-220A and SMM-100). The final recording site in each mouse was marked by an electrical microlesion (0.01 mA direct current, 60 s) (Heiney et al., 2018), and the other locations of recorded Purkinje cells were reconstructed based on the stereotaxic position of each recording location relative to the lesion site, which was visualized by Nissl staining. Recording data were excluded from further analysis if Purkinje cells were not located in lobule V. Data were recorded, and stimuli were delivered using an integrated Tucker-Davis Technologies and MATLAB system (TDT RZ5, medusa, RPVdsEx) running custom code (github.com/blinklab/neuroblinks). SSpks and CSpks were sorted offline using threshold-crossing and template-matching algorithms in Spike2 software (Cambridge Electronic Design). Experimenters blind to mouse genotype also examined voltage waveform traces throughout the recording sessions, performed additional manual sorting, and excluded recordings with poor SSpk and CSpk isolation as further quality-control measures for spike sorting. Finally, to confirm that SSpks and CSpks originated from the same Purkinje cell, we checked that there was a 10–40 ms pause in SSpk activity after each CSpk (Simpson et al., 1996). Recordings with poor SSpk and CSpk isolation were excluded from further analysis. Inter-spike intervals (ISIs) were calculated for consecutive simple spikes. CV was computed by the standard deviation of the ISIs divided by the mean of the ISIs, and CV2 was computed by 2 × |ISI_n_ – ISI_n-1_| / (ISI_n_ + ISI_n-1_) and averaged across all consecutive ISIs within a neuron.

### Western blotting

The cerebellum of KO and control mice was rapidly dissected, flash frozen in liquid nitrogen, and stored at −80°C until further use. The cerebellum was placed in a glass homogenizer with ice-cold lysis buffer (2% sodium dodecyl sulfate [SDS], 100 mM Tris–HCl, pH 7.5, protease inhibitor, and phosphatase inhibitor). The cerebellum was homogenized with 10 strokes of pestle A and 10 strokes of pestle B. Samples were sonicated on the Bioruptor sonication device (Diagendoe) for 30 s ON and 30 s OFF for 10 cycles. The homogenate was centrifuged at 10,000 × g for 10 min at 4°C. Protein concentration from the supernatant was measured using a Pierce BCA Protein Assay Kit (Thermo Fisher). Lysates were diluted to 1 μg/μl in an extraction buffer of 1× NuPAGE LDS Sample Buffer (Thermo Fisher), 1× NuPAGE Sample Reducing Agent (Thermo Fisher), and 1× RIPA buffer (100 mM Tris pH 8.0, 300 mM NaCl, 0.2% SDS, 1% sodium deoxycholate, 2% Nonidet P-40, 5 mM EDTA, protease inhibitor, and phosphatase inhibitor). Samples were then heated at 95°C for 10 min. Ten micrograms of protein was loaded into a 15-well NuPAGE 4–12% Bis–Tris gel (Thermo Fisher) and run in MES buffer (50 mM MES, 50 mM Tris base, 0.1% SDS, 1 mM EDTA pH 7.3) for 20 min at 200 V. Proteins were transferred onto PVDF blotting membranes (GE Healthcare Life Sciences) in Tris–glycine buffer (25 mM Tris, 190 mM glycine, 20% methanol). Membranes were rinsed in ddH_2_O and dried at room temp for 1 hr. Membranes were rehydrated in methanol, blocked for 1 hr at room temperature with Odyssey TBS Blocking Buffer (LI-COR Biosciences), and incubated in primary antibody diluted in blocking buffer overnight at 4°C. The following primary antibodies were used: rabbit anti-MeCP2 (1:1000, Cell Signaling Technology) and rabbit anti-Histone H3 (1:20,000, Abcam). The membranes were washed with TBS-T for 10 min, and then incubated in secondary diluted in blocking buffer for 2 hr at room temperature. The following secondary antibody was used: IRDye 680RD goat anti-rabbit IgG (LI-COR Biosciences). The membranes were washed with TBS-T for 10 min and imaged on an Odyssey imager (LI-COR Biosciences). Relative signal intensity was quantified using Image Studio Lite (LI-COR Biosciences).

### Statistical analysis

Data are displayed as mean ± s.e.m., and the significance threshold was set at α=0.05 (ns, *p<0.05, **p<0.01, ***p<0.001, ****p<0.0001). Sample sizes were determined based on prior statistics and data characterizing the phenotypes of MeCP2 mutant mice (Chao et al., 2010; Ito-Ishida et al., 2015; Meng et al., 2016). Statistical analysis was performed using Prism (GraphPad). Data were analyzed with the experimenters blinded to genotype.

## Data Availability

All data generated or analyzed during this study are included in the manuscript and supporting files.

## Additional files

Source data 1. Statistical analysis related to Figure 1, Figure 2, Figure 2—figure supplement 1, Figure 2—figure supplement 3, Figure 3, and Figure 4.

Transparent reporting form
