# Supplementary material for: Deleting Mecp2 from the cerebellum rather than its neuronal subtypes causes a delay in motor learning in mice
Source: eLife. 2021 Jan 26;10:e64833. doi: 10.7554/eLife.64833 (PMC7837679; doi:10.7554/eLife.64833)
Supplement: Peer review history

# Decision letter

**DOI:** 10.7554/eLife.64833.sa1

**Affiliations:** Oregon Health and Science UniversityUnited States; Oregon Health and Science UniversityUnited States

In the interests of transparency, eLife publishes the most substantive revision requests and the accompanying author responses.

**Acceptance summary:**

This short report describes the phenotypes observed when *Mecp2* (mutations of which cause Rett syndrome) was deleted from the entire cerebellum, but not when it was deleted from specific cerebellar cell types. The authors used a range of assays to assess the effect of deleting *Mecp2* and the experiments in general are well conducted and analyzed. Interestingly, motor learning attenuated some of the phenotypes. Although the underlying mechanism(s) are not revealed by these studies, the results provide a foundation for future work. All reviewers were pleased with your revisions.

**Decision letter after peer review:**

Thank you for submitting your article "Deleting *Mecp2* from the entire cerebellum rather than its neuronal subtypes causes a delay in motor learning in mice" for consideration by *eLife*. Your article has been reviewed by three peer reviewers, including Gail Mandel as the Reviewing Editor and Reviewer #1, and the evaluation has been overseen by Gary Westbrook as the Senior Editor.

The reviewers have discussed the reviews with one another and the Reviewing Editor has drafted this decision to help you prepare a revised submission.

As the editors have judged that your manuscript is of interest, but as described below that additional experiments are required before it is published, we would like to draw your attention to changes in our revision policy that we have made in response to COVID-19 (https://elifesciences.org/articles/57162). First, because many researchers have temporarily lost access to the labs, we will give authors as much time as they need to submit revised manuscripts. We are also offering, if you choose, to post the manuscript to bioRxiv (if it is not already there) along with this decision letter and a formal designation that the manuscript is "in revision at *eLife*". Please let us know if you would like to pursue this option. (If your work is more suitable for medRxiv, you will need to post the preprint yourself, as the mechanisms for us to do so are still in development.)

Essential revisions:

All reviewers thought this work made new and interesting points, appropriate for a short report format, related to a cerebellar contribution to the neurological disorder, Rett syndrome. The authors should address all of the comments/concerns in the three reviews as listed below, but we mention the more major ones here. None of the reviewers requested new experiments.

1) All reviewers had questions/comments, based on the cre deleter approach, about authors' interpretation of which cerebellar brain region or cell types contributed to the phenotypes under study. Responses to this issue will likely require clarification/modifications to their Discussion.

2) Two reviewers also had concerns/questions related to the physiology recordings which need to be addressed and may require, in addition to editorial clarification, new representative traces.

3) Finally, one reviewer questions the value of the gene expression changes, which lead to some open ended questions.

We hope you find these comments useful and we look forward to your revised manuscript.

Reviewer #1:

In this work, using the cre deleter system, authors delete *Mecp2* from either specific cell types within cerebellum or from "whole cerebellum", and then test for Rett-like phenotypes in the mice. For the whole cerebellum deletions, authors use the *En1* promoter to drive the cre recombinase. This cre deleter strategy follows previous successful studies by the Zoghbi lab used to uncover brain region or cell specific phenotypes contributing to Rett-like symptoms in mice. The phenotypes herein are evaluated using a multi-disciplinary approach involving genetic manipulation with immunohistochemical validation, motor behaviors, training (motor learning), electrophysiology and transcription. There are two major unexpected/interesting findings from this work: (1) only removal from the whole cerebellum results in phenotypes and (2) an improvement in motor learning by training. While potentially important and at reasonable depth for a short report, authors should clarify some of the methods or experiments that underlie their conclusions.

Figure 3. Technical point: The acquisition of the representative data presented was digitized too slowly, leading to missing pieces of the traces. Also, there appears to be something qualitatively different about the *KO* trace. When the figure is enlarged, the data seems to show more than one trace present in places (e.g. last SS). A quantitative description for the criteria by which the simple and complex traces were distinguished and resolved from one another needs to be articulated in legend and Materials and methods.

Title and Abstract: Curious why authors don't emphasize the finding that training reverses the cerebellar phenotypes because that is a very interesting aspect of the work.

The authors' descriptor of "whole" cerebellum is imprecise and could benefit from better wording, perhaps, for example, describing the result as deletion of major neuronal types in the cerebellum. I raise this issue because Figure 2—figure supplement 1 shows cre recombination (TdT positivity) from the EN1 promoter in an area adjacent to the cerebellum. This result is consistent with the JAX description of this line, cited in the Materials and methods as the source for this line, which also points to expression in tissues outside the cerebellum, including muscle. In the Discussion, authors should discuss this issue specifically along with their references to work indicating that other brain regions are not likely involved in the phenotypes measured here.

Related to above, authors confirm a lack of MeCP2 expression in specific neuronal populations in cerebellum using the *En1-Cre*, but there are other less abundant cell types in the cerebellum including oligodendroyctes and Bergman glia. Does the En1 Cre mediate excision from these populations as well? If not, could the expression of MeCP2 in these cells be preventing the appearance of a more robust phenotype? Again, this could be a discussion point.

Where there any cellular deficits following the loss of MeCP2 in individual populations? For example, several labs including that of the senior author, have described alterations in heterochromatin in MeCP2 mutant neurons. It would be interesting to see if this phenotype was present in the various cell types of the cerebellum even though there were no behavioral phenotypes.

The Hoshino et al., 2005 reference is incorrect. This paper in pub med is attributed to a different set of authors: Meredith et al. in the Johnson lab. I believe the reference should be: Hoshino M, Nakamura S, Mori K, Kawauchi T, Terao M, Nishimura YV, Fukuda A, Fuse T, Matsuo N, Sone M, Watanabe M, Bito H, Terashima T, Wright CVE, Kawaguchi Y, Nakao K, Nabeshima YI. *Ptf1a*, a bHLH transcriptional gene, defines GABAergic neuronal fates in cerebellum. Neuron. 2005;47:201-213. doi: 10.1016/j.neuron.2005.06.007.

For all figures, legends need to state the type of statistical analyses used., not just the p values. Figure 3 as noted above.

Reviewer #2:

In this manuscript the authors investigated the role of the cerebellum in Rett syndrome, characterized by loss-of-function in MECP2. MeCP2 *KO* mice in basal ganglia and cortex present severe motor impairments but cerebellar *KO* mice have not been studied. To address this question, the authors first studied rotarod performance of cerebellar cell-specific *KO* mice and whole cerebellum *KO* mice at 2, 4 and 6-month-old. They verified that the delay observed in 6-month-old cerebellar *KO* mice in motor learning was cerebellar specific and was no due to deficiency in general locomotor activity or strength. They also attest of any non-motor symptoms in these mice. They combined these behavioral experiments with electrophysiological recordings and morphological study of Purkinje cells as well as transcriptional analysis of the whole cerebellum.

They concluded that cerebellar dysfunction (more variability of firing pattern of Purkinje cells and transcriptional abnormalities) is involved somehow in the motor deficiencies observed in MeCP2 *KO* mice. Overall, this is a very nice piece of work. Nevertheless, I have some comments below that I feel will help improve the clarity of the paper.

1) One of the major finding of the paper is that *KO* in different subpopulations does not cause deficiency; however, there is no real *KO* specific for inhibitory interneurons of the cerebellum (both Purkinje and interneurons (Figure 2—figure supplement 2G-I). The conclusion may not be appropriate; please discuss.

2) Why recordings of PC only? When it is shown that it is not the only population involved in that deficit as MeCP2 *KOL7* mice show no deficits. Please justify. Also, there is no CV measurement? Why only CV2?

3) Complete depletion of *Mecp2* causes ataxia in mice. But complete depletion of *Mecp2* in the cerebellum of mice does not induce ataxia, which is mainly caused by cerebellar dysfunction. Please discuss.

4) Are the 2-month-old, 4-month-old, and 6-month-old mice the same animals?

Reviewer #3:

The study presented a clear question to be investigated, used appropriate paradigms to address the question, and the results are largely clear. Sample sizes are more than sufficient given the variances reported, and the statistical analyses employed appropriate stringent tests. The study does selectively focus on male subjects, but given the specific question being asked, and the confound X-linked mosaicism would have on subtle behavioral changes in females, this choice is defendable. In short, this is a strong study overall and I have only a few suggestions for the authors to consider.

The first relates to the specificity of cre expression in the driver lines employed. What was shown nicely was the near complete ablation of MeCP2 in total cerebellum or in the targeted cell populations of the cerebellum. What is not as clear is to what degree (if any) recombination occurred within cells in other regions of the brain. While *En1-Cre* is largely restricted to cerebellum, *Atoh1-Cre* and *Ptf1a-Cre* are also expressed peripherally and in other brain regions. Figure 2—figure supplement 2 shows preservation of MeCP2 hippocampus in the *En1-Cre* line, which is not unexpected, but there is no discussion about non-cerebellar targets. The low magnitude view of Td-tomato is insufficient to show modest expression levels in extra-cerebellar cells. To be fair, this does not affect the results interpretations as none of the cell-type specific ablations yielded phenotypic alterations. But for at least *Pftf1-Cre* mice, recombination would be expected in a host of other interneuronal populations, and the lack of behavioral consequence would indicate the ablation of *Mecp2* from those targets is also insufficient for behavioral manifestations (which is consistent with previous work from the group).

The second relates to the behavioral tasks selected and the degree to which cerebellum function influences performances in those tasks. The tasks interrogated are a standard informative battery, but do not include fine dexterity assays that would be more likely to reveal a selective dysfunction of cerebellar circuitry. The conditioned eye blink test is arguably the most cerebellar sensitive. While performance delays were seen in the cerebellar-*KO* mice relative to Flox mice, they did "learn" the task and reach the probability and amplitude responses of the Flox mice. However, unlike the rotarod test, there did not appear to be any increased learning rate. Might this suggest differences in how internal cerebellar circuits are affected the absence of *Mecp2*?

The third relates to the candidate gene expression comparisons. It is not clear what this section adds to the study mechanistically without additional investigation of candidate gene product function. Further, the results did not reproduce the changes previously reported for 16 of the 20 targets investigated, and this was also not discussed. The progressiveness of alteration shown for the 4 targets was also interesting, but not discussed. Why would preservation be seen at 2 months? The postnatal maturation of the cerebellum is largely complete by 2 months, and the lack of changes at this time indicates these alterations do not simply arise from MeCP2 absence. This section would benefit from additional discussion or it could be removed and serve as the starting point for an subsequent study.

# Reply: Author response

**DOI:** 10.7554/eLife.64833.sa2

Reviewer #1:

In this work, using the cre deleter system, authors delete Mecp2 from either specific cell types within cerebellum or from "whole cerebellum", and then test for Rett-like phenotypes in the mice. For the whole cerebellum deletions, authors use the En1 promoter to drive the cre recombinase. This cre deleter strategy follows previous successful studies by the Zoghbi lab used to uncover brain region or cell specific phenotypes contributing to Rett-like symptoms in mice. The phenotypes herein are evaluated using a multi-disciplinary approach involving genetic manipulation with immunohistochemical validation, motor behaviors, training (motor learning), electrophysiology and transcription. There are two major unexpected/interesting findings from this work: (1) only removal from the whole cerebellum results in phenotypes and (2) an improvement in motor learning by training. While potentially important and at reasonable depth for a short report, authors should clarify some of the methods or experiments that underlie their conclusions.

Figure 3. Technical point: The acquisition of the representative data presented was digitized too slowly, leading to missing pieces of the traces. Also, there appears to be something qualitatively different about the KO trace. When the figure is enlarged, the data seems to show more than one trace present in places (e.g. last SS). A quantitative description for the criteria by which the simple and complex traces were distinguished and resolved from one another needs to be articulated in legend and Materials and methods.

We apologize for the poor image quality. We replaced them with high-resolution images. We included a brief description of the criteria for simple and complex spikes in the legend and included a detailed description in the Materials and methods.

Title and Abstract: Curious why authors don't emphasize the finding that training reverses the cerebellar phenotypes because that is a very interesting aspect of the work.

This is indeed an interesting finding and was the inspiration for a much more expanded story that is beyond the scope of this study. We included this piece of data here to provide a complete picture of our findings, however, because the data are limited to males and we did not perform additional experiments to follow up on this observation, we did not feel that including it in the title was appropriate.

The authors' descriptor of "whole" cerebellum is imprecise and could benefit from better wording, perhaps, for example, describing the result as deletion of major neuronal types in the cerebellum. I raise this issue because Figure 2—figure supplement 1 shows cre recombination (TdT positivity) from the EN1 promoter in an area adjacent to the cerebellum. This result is consistent with the JAX description of this line, cited in the Materials and methods as the source for this line, which also points to expression in tissues outside the cerebellum, including muscle. In the Discussion, authors should discuss this issue specifically along with their references to work indicating that other brain regions are not likely involved in the phenotypes measured here.

Rather than saying “entire cerebellum” we now say “all major neuronal subtypes in the cerebellum” when describing the results. We also discuss the issue of Cre specificity in the Discussion. Although *En1* is expressed in the midbrain and spinal cord interneurons, we do not believe this contributes to the phenotypes we observed because dopaminergic (*TH-Cre*) *KO* mice have a normal rotarod performance (Samaco et al., 2009). These mice lack *Mecp2* in the substantia nigra, a region in the midbrain that is critical for motor function. Thus, it is less likely that midbrain dysfunction contributes to the rotarod deficit we observed in our *En1-Cre KO* mice. Although, the loss of *Mecp2* from spinal cord interneurons could contribute to the rotarod deficits, they are unlikely to affect eyeblink conditioning because this reflex is mediated by circuits in the cerebellum and brainstem (Bracha, 2004). Finally, the loss of *Mecp2* from skeletal muscle does not affect muscle morphology or physiology (Conti et al., 2015), so *En1-Cre* expression and the subsequent removal of *Mecp2* in muscle is unlikely to contribute to the motor phenotypes we observed.

Related to above, authors confirm a lack of MeCP2 expression in specific neuronal populations in cerebellum using the En1-Cre, but there are other less abundant cell types in the cerebellum including oligodendroyctes and Bergman glia. Does the En1-Cre mediate excision from these populations as well? If not, could the expression of MeCP2 in these cells be preventing the appearance of a more robust phenotype? Again, this could be a discussion point.

*En1-Cre* mediates excision from cerebellar oligodendrocytes and Bergmann glia because these are derived from progenitors in rhombomere 1 (part of the *En1* expression domain) (Hashimoto et al., 2016; Yamada and Watanabe, 2002).

Where there any cellular deficits following the loss of MeCP2 in individual populations? For example, several labs including that of the senior author, have described alterations in heterochromatin in MeCP2 mutant neurons. It would be interesting to see if this phenotype was present in the various cell types of the cerebellum even though there were no behavioral phenotypes.

We analyzed heterochromatin architecture by staining for DAPI, H3K4me3, H3K9me3, and H3K27me3 in cerebellar *KO* mice as well as mice lacking *Mecp2* in cerebellar subtypes. The amount of H3K9me3 in the heterochromatic foci of granule cells, Purkinje cells, and molecular layer interneurons was reduced in cerebellar *KO* mice compared to control mice. Interestingly, this defect was also present in granule cells of *Atoh1-Cre KO* mice, Purkinje cells of *L7-Cre KO* mice, and Purkinje cells and molecular layer interneurons of *Ptf1a-CreKO*. These findings are described in a new Figure 4 since to replace the transcriptional data (old Figure 4) that reviewer 3 felt were not necessary.

The Hoshino reference is incorrect. This paper in pub med is attributed to a different set of authors: Meredith et al. in the Johnson lab. I believe the reference should be: Hoshino M, Nakamura S, Mori K, Kawauchi T, Terao M, Nishimura YV, Fukuda A, Fuse T, Matsuo N, Sone M, Watanabe M, Bito H, Terashima T, Wright CVE, Kawaguchi Y, Nakao K, Nabeshima YI. Ptf1a, a bHLH transcriptional gene, defines GABAergic neuronal fates in cerebellum. Neuron. 2005;47:201-213. doi: 10.1016/j.neuron.2005.06.007.

Thank you for this important catch. It is now corrected.

For all figures, legends need to state the type of statistical analyses used., not just the p values. Figure 3 as noted above.

We added the type of statistical analyses used to each figure legend.

Reviewer #2:

In this manuscript the authors investigated the role of the cerebellum in Rett syndrome, characterized by loss-of-function in MECP2. MeCP2 KO mice in basal ganglia and cortex present severe motor impairments but cerebellar KO mice have not been studied. To address this question, the authors first studied rotarod performance of cerebellar cell-specific KO mice and whole cerebellum KO mice at 2, 4 and 6-month-old. They verified that the delay observed in 6-month-old cerebellar KO mice in motor learning was cerebellar specific and was no due to deficiency in general locomotor activity or strength. They also attest of any non-motor symptoms in these mice. They combined these behavioral experiments with electrophysiological recordings and morphological study of Purkinje cells as well as transcriptional analysis of the whole cerebellum.

They concluded that cerebellar dysfunction (more variability of firing pattern of Purkinje cells and transcriptional abnormalities) is involved somehow in the motor deficiencies observed in MeCP2 KO mice. Overall, this is a very nice piece of work. Nevertheless, I have some comments below that I feel will help improve the clarity of the paper.

1) One of the major finding of the paper is that KO in different subpopulations does not cause deficiency; however, there is no real KO specific for inhibitory interneurons of the cerebellum (both Purkinje and interneurons (Figure 2—figure supplement 2G-I). The conclusion may not be appropriate; please discuss.

Although there is no *Cre*-expressing mouse that exclusively targets molecular layer interneurons of the cerebellum, we still conclude that the loss of *Mecp2* in this population does not cause behavioral deficits. Because *L7-CreKO* mice do not have behavioral deficits, the lack of behavioral deficits in *Ptf1a-Cre KO* mice indicates that deleting *Mecp2* from molecular layer interneurons is also not sufficient to cause behavioral deficits. We clarified this in the Discussion.

2) Why recordings of PC only? When it is shown that it is not the only population involved in that deficit as MeCP2 KO L7 mice show no deficits. Please justify. Also, there is no CV measurement? Why only CV2?

There are two main reasons why we chose to study Purkinje cells in our in vivo analyses. First, granule cell parallel fibers and molecular layer interneuron axons together modulate simple spike activity, and inferior olivary neurons drive complex spikes. Recording from Purkinje cells is an efficient and reliable method of examining the entire circuit in vivo because they serve as the final output stage of the cerebellar cortex, so we can acquire information about the entire circuit from a single cell type. Second, recording from the other cell types in the cerebellar cortex is more challenging, as their signals are more ambiguous and less reliable. Granule cell signals are complex and difficult to isolate in vivo due to their long periods of silence (Ruigrok, Hensbroek and Simpson, 2011). Although molecular layer interneurons can be recorded in vivo (Ruigrok, Hensbroek and Simpson, 2011; Barmack and Yakhnitsa, 2008), their sparse distribution and “gradient” of identities (stellate cells or basket cells) makes analyzing their function more reliable when they are analyzed indirectly as changes in Purkinje cell simple spike activity (Brown et al., 2019). Therefore, Purkinje cells are the ideal cell type for examining whether functional changes have occurred at any point in the cerebellar circuit.

We included CV measurements in Figure 3.

3) Complete depletion of Mecp2 causes ataxia in mice. But complete depletion of Mecp2 in the cerebellum of mice does not induce ataxia, which is mainly caused by cerebellar dysfunction. Please discuss.

Previous work has shown that complete loss of *Mecp2* causes ataxia, as revealed by an impairment on the rotarod (Pelka et al., 2006). We have shown that the loss of *Mecp2* in the cerebellum also cause a similar impairment. In contrast to the permanent impairments observed in global *Mecp2KO* mice, however, the impairments in the cerebellar *KO* mice improved with additional training. We modified the text in the Introduction and Discussion to clarify similarities and differences between our observations and those previously reported in global *KO* mice. We hypothesize that the motor impairments on the rotarod in global *Mecp2KO* arise from dysfunction in the cerebellum as well as other regions implicated in motor function, such as the cortex and basal ganglia.

4) Are the 2-month-old, 4-month-old, and 6-month-old mice the same animals?

These are different cohorts of animals. This was clarified in the legend of Figure 2—figure supplement 3.

Reviewer #3:

The study presented a clear question to be investigated, used appropriate paradigms to address the question, and the results are largely clear. Sample sizes are more than sufficient given the variances reported, and the statistical analyses employed appropriate stringent tests. The study does selectively focus on male subjects, but given the specific question being asked, and the confound X-linked mosaicism would have on subtle behavioral changes in females, this choice is defendable. In short, this is a strong study overall and I have only a few suggestions for the authors to consider.

The first relates to the specificity of cre expression in the driver lines employed. What was shown nicely was the near complete ablation of MeCP2 in total cerebellum or in the targeted cell populations of the cerebellum. What is not as clear is to what degree (if any) recombination occurred within cells in other regions of the brain. While En1-Cre is largely restricted to cerebellum, Atoh1-Cre and Ptf1a-Cre are also expressed peripherally and in other brain regions. Figure 2—figure supplement 2 shows preservation of MeCP2 hippocampus in the En1-Cre line, which is not unexpected, but there is no discussion about non-cerebellar targets. The low magnitude view of Td-tomato is insufficient to show modest expression levels in extra-cerebellar cells. To be fair, this does not affect the results interpretations as none of the cell-type specific ablations yielded phenotypic alterations. But for at least Pftf1-Cre mice, recombination would be expected in a host of other interneuronal populations, and the lack of behavioral consequence would indicate the ablation of Mecp2 from those targets is also insufficient for behavioral manifestations (which is consistent with previous work from the group).

Indeed, the loss of *Mecp2* in inhibitory neurons plays an important role in the pathogenesis of Rett syndrome phenotypes (Chao et al., 2010; Ito-Ishida et al., 2015). However, cerebellar interneurons are the only inhibitory neurons within the brain that express *Ptf1a* (Meredith et al., 2009). *Ptf1a* is also expressed outside the cerebellum in neurons of the inferior olive (Hoshino et al., 2005), which send inputs onto Purkinje cells via climbing fibers, and spinal cord interneurons (Bikoffet al., 2016). Although our study focuses on the cerebellum, we can conclude that the loss of *Mecp2* in these extracerebellar regions is also not sufficient to cause behavioral phenotypes.

The second relates to the behavioral tasks selected and the degree to which cerebellum function influences performances in those tasks. The tasks interrogated are a standard informative battery, but do not include fine dexterity assays that would be more likely to reveal a selective dysfunction of cerebellar circuitry. The conditioned eye blink test is arguably the most cerebellar sensitive. While performance delays were seen in the cerebellar-KO mice relative to Flox mice, they did "learn" the task and reach the probability and amplitude responses of the Flox mice. However, unlike the rotarod test, there did not appear to be any increased learning rate. Might this suggest differences in how internal cerebellar circuits are affected the absence of Mecp2?

Differences in the learning rates of rotarod and eyeblink conditioning may reflect how internal cerebellar circuits respond to the absence of *Mecp2.* How these circuits adapt to training in cerebellar *KO* mice is an area of ongoing investigation in our laboratory.

The third relates to the candidate gene expression comparisons. It is not clear what this section adds to the study mechanistically without additional investigation of candidate gene product function. Further, the results did not reproduce the changes previously reported for 16 of the 20 targets investigated, and this was also not discussed. The progressiveness of alteration shown for the 4 targets was also interesting, but not discussed. Why would preservation be seen at 2 months? The postnatal maturation of the cerebellum is largely complete by 2 months, and the lack of changes at this time indicates these alterations do not simply arise from MeCP2 absence. This section would benefit from additional discussion or it could be removed and serve as the starting point for an subsequent study.

We removed this section from the manuscript as it will serve as the foundation for a follow-up study. We replaced it with chromatin architecture experiments (new Figure 4) that were requested by reviewer #1.

References:

1) Barmack NH, Yakhnitsa V. 2008. Functions of interneurons in mouse cerebellum. J Neurosci 28:1140-52. doi: 10.1523/JNEUROSCI.3942-07.2008

2) Brown AM, Arancillo M, Lin T, Catt DR, Zhou J, Lackey EP, Stay TL, Zuo Z, White JJ, Sillitoe RV. 2019. Molecular layer interneurons shape the spike activity of cerebellar Purkinje cells. Sci Rep 9:1742. doi: 10.1038/s41598-018-38264-1

3) Meredith DM, Masui T, Swift GH, MacDonald RJ, Johnson JE. 2009. Multiple transcriptional mechanisms control *Ptf1a* levels during neural development including autoregulation by the PTF1-J complex. J Neurosci 29:11139-48. doi: 10.1523/JNEUROSCI.2303-09.2009

4) Ruigrok TJ, Hensbroek RA, Simpson JI. 2011. Spontaneous activity signatures of morphologically identified interneurons in the vestibulocerebellum. J Neurosci 31:712-24. doi: 10.1523/JNEUROSCI.1959-10.2011

5) Yamada K, Watanabe M. 2002. Cytodifferentiation of Bergmann glia and its relationship with Purkinje cells. Anat Sci Int 77:94-108. doi: 10.1046/j.0022-7722.2002.00021.x
